# Finite Element Analysis of Gas Diffusion in Polymer Nanocomposite Systems Containing Rod-like Nanofillers

**DOI:** 10.3390/polym13162615

**Published:** 2021-08-06

**Authors:** Thouaiba Htira, Sarra Zid, Matthieu Zinet, Eliane Espuche

**Affiliations:** Ingénierie des Matériaux Polymères, University Lyon, Université Lyon 1, CNRS UMR 5223, F-69622 Villeurbanne, France; htira.thouaiba@hotmail.fr (T.H.); sarrabz.ing@gmail.com (S.Z.)

**Keywords:** diffusion, modeling, cellulose nanocrystals, barrier properties, interphase

## Abstract

Polymer-based films with improved gas barrier properties are of great interest for a large range of applications, including packaging and coatings. The barrier effect is generally obtained via the addition of a sufficient amount of impermeable nanofillers within the polymer matrix. Due to their low environmental footprint, bio-based nanocomposites such as poly(lactic acid)–cellulose nanocrystal (PLA–CNC) nanocomposites seem to be an interesting alternative to synthetic-polymer-based nanocomposites. The morphology of such systems consists of the dispersion of impermeable rod-like fillers of finite length in a more permeable matrix. The aim of this work is to analyze, through finite element modeling (FEM), the diffusion behavior of 3D systems representative of PLA–CNC nanocomposites, allowing the determination of the nanocomposites’ effective diffusivity. Parametric studies are carried out to evaluate the effects of various parameters, such as the filler volume fraction, aspect ratio, polydispersity, and agglomeration, on the improvement of the barrier properties. The role of the filler–matrix interfacial area (or interphase) is also investigated and is shown to be particularly critical to the overall barrier effect for highly diffusive interphases.

## 1. Introduction

Polymer-based films with improved gas barrier properties are of great interest for a large range of applications, including packaging (for food or other goods) or coatings. In past decades, petroleum-based polymers were commonly used for such applications due to their ease of processing, interesting mechanical properties, and low cost. Numerous studies have shown that the barrier properties of these materials can be greatly improved by adding impermeable lamellar nanofillers, such as montmorillonite, layered double hydroxides (LDH), or zirconium phosphate [1,2,3,4,5,6,7]. Indeed, based on the path length theory, the barrier properties in such binary systems are improved by increasing the distance to travel in the polymer matrix, which are then strongly related to the aspect ratio of the fillers and their dispersion state [8,9,10]; however, such materials still have a high environmental impact. With these concerns in mind, growing interest has been paid to sustainable materials. The development of green materials has been widely investigated in recent decades. In this context, due to their low environmental footprint, bio-based polymers seemed to be a good alternative to synthetic polymers. Among these, poly(lactic acid) (PLA) is currently one of the best candidates. Polymerized from lactic acid monomers extracted from agricultural products (corn, beet, sugar cane), PLA exhibits very interesting properties, such as transparency, high stiffness, printability, ease of processing, recyclability, and biodegradability in compost [11,12,13]; however, such bio-based polymers generally demonstrate low barrier properties compared to commonly derived petroleum polymers [14], which could limit their attractiveness in the packaging industry. One strategy to increase the barrier level of these polymers while maintaining their sustainability should be to introduce very low-permeable bio-based domains within these matrices. For this purpose, cellulose-based nanomaterials have great potential as renewable and sustainable materials. They are extracted from various cellulosic sources such as wood, cotton, flax, sisal, or algae using different routes, essentially leading to two types of nanofillers: cellulose nanofibrils (CNFs) [15,16,17] and cellulose nanocrystals (CNCs) [18,19,20]. Due to their excellent properties such as their high crystallinity, low density, high specific surface area, and high surface reactivity, CNFs and CNCs have been involved in numerous innovative applications requiring bio-based or biodegradable materials [21,22,23], and more specifically with the purpose of developing PLA-based nanocomposites [24,25,26]. The surface functionalization of the cellulosic nanostructures (often via grafting routes) is generally a required step in order to achieve good adhesion with the polymeric matrix [19,27,28]. 

Although CNCs are more difficult to produce than CNFs, their higher crystallinity, shorter length, and lower propensity to agglomerate [29] make them the most interesting choice for a number of applications, including food packaging. Regarding their intrinsic gas barrier properties, CNCs present themselves as rod-like particles with two nanoscale dimensions and O_2_ and CO_2_ diffusion coefficients reported to be of an order of magnitude of 10^−15^ m^2^/s [30], which is much lower than O_2_ and CO_2_ diffusion coefficients in a PLA matrix (in the order of magnitude of 10^−12^ m^2^/s).

Different experimental studies have investigated the effects of combining CNCs with polymer matrices on barrier properties. Nuruddin et al. [31] obtained an increase of barrier properties by 13% by adding 30 wt% CNCs in a polyvinyl alcohol (PVA) matrix. In this study, the length and width of CNCs were 100 ± 25 and 5 ± 1.5 nm, respectively. These authors also showed that depending on the PVA content, anisotropic or isotropic nanocomposite films were obtained. Rader at al. [32] reported oxygen transmission rate (OTR) reductions of as much as 40% for a CNC content of 10 wt% within a bio-based polyester-amide (PEA) matrix. It was shown that the barrier enhancement was highly dependent on the composition of the PEA matrix but much less so on the CNC content. For the same CNC content, Mondragon et al. [33] obtained a 37% decrease of OTR for a gelatin matrix. In this case, the CNCs had a length-to-diameter ratio equal to 53. Bendhaou et al. [34] reached a relative OTR value of 0.33 by introducing 5 wt% CNC in a natural rubber. This high increase of barrier properties was attributed to a specific morphology of the nanocomposites. Poly(3-hydroxybutyrate) (PHB)–CNC-based nanobiocomposite films were investigated by Dhar and coworkers [35]. A decrease of oxygen diffusion of 17% was obtained by the authors with loading fractions similar to 2 wt% due to the hydrogen-bonded interaction of PHB with CNCs, as well as the tortuous path provided towards oxygen permeation. From these literature data, it can clearly be seen that numerous parameters such as nanocomposite morphology and polymer–CNC interfacial properties can play significant roles in the resulting barrier properties. 

One way to better understand the barrier properties of such systems is to perform step-by-step modeling of the nanocomposite behavior as a function of parameters such as the nanorod amount, nanorod dispersion state, or nanorod–polymer interfacial properties. It must be noted that although a lot of work has been devoted to modeling barrier properties of nanocomposites containing spheres and lamellar nanofillers, to our knowledge, no detailed modeling approach has been focused on nanocomposites containing rod-like nanofillers. The literature concerning the available analytical models and numerical approaches for estimation of the effective diffusivity in multiphase polymer systems has been extensively reviewed by Zid et al. [36]. One of the first models describing the diffusion behavior of a single component (gas) in a heterogeneous media (matrix filled with spheres) was proposed by Maxwell, who proposed a simple expression for the prediction of the effective diffusion coefficient of a heterogeneous media functioning in the dispersed phase volume fraction [37]. Then, models considering ribbons of infinite length were developed and other shapes, such as disks in both 2 and 3 dimensions, were also investigated. Despite the number of existing models, there is still a gap in the development of 3D models, which are indispensable for predicting the diffusion behavior of systems filled with randomly dispersed finite-length objects, such as spheres, flakes, and cylinders. 

The aim of this paper is to analyze, through numerical modeling, the diffusion behavior of gases in three-dimensional model systems consisting of a polymer matrix filled with randomly dispersed rod-like nanocrystals of finite length. The key parameters of these systems are chosen to be representative of bio-based polymer–CNC nanocomposites. The modeling approach is based on the assumption of Fickian diffusion, which generally governs the gas transport properties in polymer-based materials and which means that the polymer matrix is assimilated to a continuous medium and that the size of the diffusing species remains small with respect to the characteristic dimensions of the medium. The implemented models are then solved with the finite element method (FEM), allowing the determination of the nanocomposite’s effective diffusivity. Parametric analyses are carried out to evaluate the effects of various characteristics of the system, such as the filler volume fraction, aspect ratio, polydispersity, and agglomeration, on the improvement of the barrier properties. The role of the filler–matrix interfacial area is also investigated through the addition of a third phase (the “interphase”) to the model. Since the interphase transport properties may vary from nearly impermeable to highly diffusive, the resulting nanocomposite barrier properties may be impacted in opposite ways. The developed model can help to understand and predict this relationship.

## 2. Materials and Methods

### 2.1. Materials 

Under the assumption of steady-state Fickian diffusion, the transport behavior of a given species in the composite system is completely governed by the diffusion coefficient of the species in each phase and by the morphology (i.e., the geometry) of these phases. This study was oriented towards simulating the diffusion of small molecules such as O_2_ and CO_2_ in PLA–CNC nanocomposites. For the PLA matrix, a single diffusion coefficient value *D*_0_ = 10^−12^ m^2^/s was assumed, which corresponds to the order of magnitude reported for the diffusion coefficients of O_2_ as well as of CO_2_ in PLA [38,39]. The CNCs were considered impermeable to the diffusing species, which is a reasonable assumption since actual diffusion coefficients of O_2_ and CO_2_ in cellulose nanocrystals have been reported to be of an order of magnitude of 10^−15^ m^2^/s [30]. Indeed, preliminary simulations have shown that taking into account the permeability of CNCs versus considering them impermeable led to a deviation in the effective nanocomposite diffusivity inferior to 0.1%. The characteristic dimensions of CNCs reported in the literature can vary across a wide range (diameter between 3 and 70 nm and length between 25 and 3000 nm), depending on the CNC source and method of observation [40]. In the present work, the CNCs were assumed to have a cylindrical (rod-like) shape, with a constant diameter *D* of 35 nm and lengths *L* ranging between 50 and 250 nm to account for length polydispersity. Defining the filler aspect ratio *α* as *L*/*D* leads to aspect ratio values ranging between 1.43 and 7.14. The CNC volume fraction was in the range of 0–35%, meaning that the nanofillers composed the dispersed phase of the nanocomposite, whereas PLA constituted the continuous phase. 

### 2.2. Geometry Modeling

A modeling methodology similar to that applied for disk-like fillers by Zid et al. [41,42] was implemented. A three-dimensional parallelepipedic representative volume element (RVE) was chosen to model the geometry of the nanocomposite in a Cartesian coordinate system (*x*,*y*,*z*), with *z* being the axis parallel to the overall diffusion direction. The dimensions of the RVE are *L_x_*, *L_y_*, and *L_z_*. The RVE is composed of at least two phases: the matrix and the fillers. A third phase called the “interphase” can be added in order to account for the filler–matrix interfacial diffusive phenomena. If present, this phase surrounds each filler and is characterized by a homogenous thickness *e_int_* and a distinct mass diffusion coefficient *D_int_*. 

**Figure 1:**
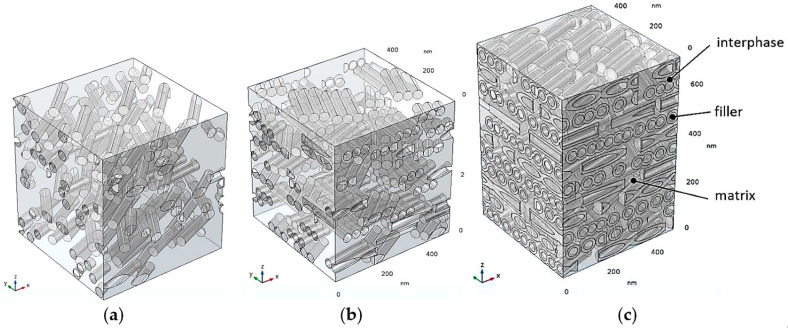
Examples of generated RVEs for the different types of model systems investigated: (**a**) 2-phase model with isolated fillers; (**b**) 2-phase model with agglomerated fillers; (**c**) 3-phase model with filler–matrix interphase areas.

The nanofiller dispersion was generated using a “layer-by-layer” approach along the *z*-axis, i.e., each layer is parallel to the *x*-*y* plane. Within a given layer, the number of fillers is determined according to the target volume fraction, then each cylinder is randomly positioned taking into account a non-overlapping condition. Note that in the case of 3-phase simulations, interphases are allowed to overlap (i.e., merge). In the case of agglomerated fillers, clusters of three identical parallel contacting rods are generated for each random position. All fillers belonging to a given layer have the same orientation in the *x*-*y* plane. The orientation angle is randomly chosen and is different from one layer to another. This is a simplified assumption, since in the actual systems, individual fillers can be randomly oriented with respect to the *x*,*y* and *z* axes; however, the conditions imposed for the present model geometries provide the upper limit case for improvements of barrier properties (all fillers perpendicular to the overall diffusive flux and increased packing in each layer, which will allow higher filler volume fractions to be reached more easily). Figure 1 shows examples of generated RVEs for the types of systems studied in this work. 

The optimal size of the RVE was determined from preliminary simulations of size-independency of the solution with varying *L_x_* (=*L_y_*) and *L_z_*. In order to minimize the computational cost and time, the objective was to determine the smallest RVE that still ensured reasonable consistency of the solution for several generated random filler dispersions with the same characteristics (volume fraction, filler aspect ratio). Moreover, for this optimal size, the solution should not vary significantly when one of the RVE dimensions is increased further. 

According to these requirements, the optimal RVE dimensions were determined as *L_x_* = *L_y_* = 500 nm and *L_z_* = 525 nm, with 15 layers of fillers along the *z* direction. For such a RVE size and a 10% volume fraction, the maximum deviation in calculated overall effective diffusivity for several equivalent random dispersions does not exceed 0.5%. Note that the actual RVE dimensions could be varied slightly around these values if required for practical reasons.

### 2.3. Governing Equations and Boundary Conditions

The diffusive mass transport equation without a mass source (Fick’s second law) was used to model the diffusion process in the nanocomposite system. In the stationary regime state, the equation reads as follow:

(1) $$\nabla \cdot \left(-D_{ij}\vec{\nabla }c_{i}\right)=0$$

with $c_{i}$ (mol·m^−3^) being the molar concentration of the permeating species *i* and $D_{ij}$ (m^2^·s^−1^) being the mass diffusion coefficient of the permeating species *i* in medium *j*. This equation was solved in the domain corresponding to the polymer matrix (with $D_{ij}=D_{0}$), while in the case of simulations accounting for filler matrix interphases, in the domains representing the interphases (with $D_{ij}=D_{int}$); however, the equation was not solved within the domains representing the fillers themselves, since they can quite reasonably be considered impermeable with respect to the matrix diffusivity.

**Figure 2:**
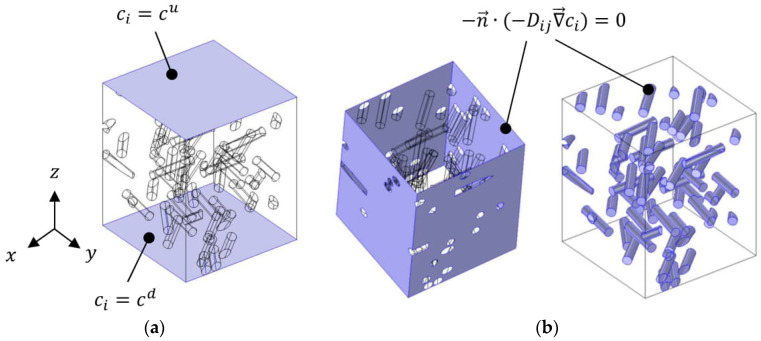
Boundary conditions applied on the RVE: (**a**) concentration BCs; (**b**) no-flux BCs.

The mass diffusion equation is complemented with the following set of boundary conditions (BCs) (Figure 2). In order to generate an overall concentration gradient along the *z*-direction, fixed concentration boundary conditions are applied on the lower and upper boundaries of the RVE:

(2) $$c_{i}\left(x,y,z=0\right)=c^{d}$$

and

(3) $$c_{i}\left(x,y,z=L_{z}\right)=c^{u}$$

According to the assumption that the matrix and interphase area diffusivities are not concentration-dependent, the values chosen for $c^{u}$ and $c^{d}$ have no influence on the nanocomposite’s effective diffusivity results; hence, the values $c^{d}$ = 1000 mol·m^−3^ and $c^{u}$ = 500 mol·m^−3^ are arbitrarily imposed. The sign of the overall diffusive flux with respect to the *z*-axis is then positive. On the lateral boundaries of the RVE, a symmetry condition is considered. On the surfaces of the fillers in contact with either the matrix (2-phase model) or the interphase area (3-phase model), impermeability is assumed. Both physical conditions are mathematically modeled by a no-flux condition:

(4) $$-\vec{n}\cdot \left(-D_{ij}\vec{\nabla }c_{i}\right)=0$$

where $\vec{n}$ is the outward normal unit vector associated with the considered surface. In the case of the 3-phase model, the internal boundary condition at the matrix–interphase boundary is simply a continuity condition for equality of the concentration and equality of the diffusive flux vector at both sides of the boundary. 

### 2.4. Numerical Analysis

**Figure 3:**
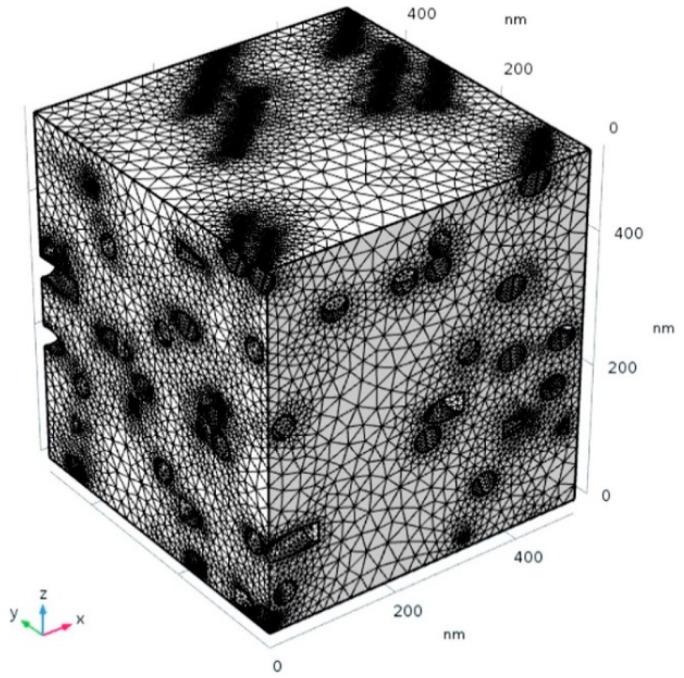
Example of a generated FE mesh of the RVE (volume fraction ≅ 10%).

The model was implemented and solved using the COMSOL Multiphysics v5.4 finite element package. In the finite element solution process used here, the computational domain is discretized according to an unstructured mesh composed of four-node tetrahedral elements. In each element, the concentration field is described as a linear interpolation of the node values (1st order elements). The mesh was automatically refined in the regions surrounding the fillers in order to improve accuracy in these regions. The total number of elements varies with the filler volume fraction. The average elemental quality index is about 0.6 (with a value of 1 corresponding to maximum quality, i.e., all elements being equilateral tetrahedra). An example of a generated mesh for a system with a volume fraction of about 10% is shown in Figure 3.

The numerical solution to the boundary value problem yields the molar concentration field of the diffusing species $c_{i}\left(x,y,z\right)$ over the computational domain. The mass flux vector $\vec{N_{i}}$ (mol·m^−2^·s^−1^) of the permeating species *i* is calculated from the concentration field as:

(5) $$\vec{N_{i}}\left(x,y,z\right)=-D_{ij}\vec{\nabla }c_{i}\left(x,y,z\right)$$

The average mass flux of the diffusing species across a plane section *S* of the RVE located at a given position *z* = *z*_0_ and normal to the *z*-direction is then given by:

(6) $$\bar{N_{i,z}}=\frac{1}{L_{x}L_{y}}\iint_{S}N_{i,z}\left(x,y,z_{0}\right)dxdy$$

where $N_{i,z}$ is the *z*-component of the mass flux vector. Finally, the effective diffusivity of the nanocomposite system is calculated by:

(7) $$D_{eff}=\frac{\bar{N_{i,z}}L_{z}}{c^{d}-c^{u}}$$

One of the most convenient parameters used to characterize and compare the enhancement of barrier properties of various systems with respect to the neat matrix properties is the relative effective diffusivity, defined as the ratio $D_{eff}/D_{0}$. In the present case, $D_{0}$ is supposed to be independent of the diffusing species concentration, which in turn implies that the relative effective diffusivity does not depend on the neat matrix diffusivity value. In the following section, the overall nanocomposite diffusivity results will be presented in terms of the relative effective diffusivity. 

## 3. Results and Discussion

### 3.1. Effects of Filler Volume Fraction

For a given filler shape, the structural parameter with the most significant influence on the barrier properties of the nanocomposite is usually the filler volume fraction. In order to study this particular relationship in the case of the nanorod-filled composite, several RVEs with volume fraction values ϕ up to 32% were generated and their effective diffusivity values were determined using the finite element simulation approach described in Section 2. The filler size distribution is monodisperse, with *D* = 35 nm and *L* = 177 nm, which correspond to an aspect ratio value *α* = 5. The calculated relative effective diffusivity values were compared to the predictions of several analytical models from the literature. Table 1 summarizes the equations of the effective relative diffusivity according to these models. 

The selected equations are either for the filler of elongated form, such as fibers and ribbons or equations depending only on the filler loading content (Maxwell [43], Bruggeman [44], Rayleigh [45], Shen and Springer [46]). Note that the Rayleigh and Maxwell models are normally limited to very low-volume fraction values. Models depending on a “slit shape” parameter (such as the work of Cussler and coworkers [47]) and models considering disk-like fillers were not used for comparison with our results. Indeed, the slit shape parameter has no significance in the case of random disposition of fillers. Moreover, the aspect ratio and the barrier effect of disk-like fillers are too different from that of rod-like fillers to provide relevant comparisons or predictions. For equations depending on both the filler volume fraction and aspect ratio, one must be careful about which definition of the aspect ratio is to be considered:

- The Nielsen model defines the aspect ratio as the ratio of the filler length to the filler width (adjusted to the rod diameter in the present case), meaning the aspect ratio value *α* = 5 should be used in the equation;
- The model’s used by Sorrentino et al. [48] and Lape et al. [49] use an alternative aspect ratio definition, namely the ratio of the shorter dimension encountered in the diffusion direction to the filler thickness. For rods perpendicular to the diffusion direction, both parameters are adjusted to the rod diameter, meaning the aspect ratio value *β* = 1 should be considered in these models.

**Figure 4:**
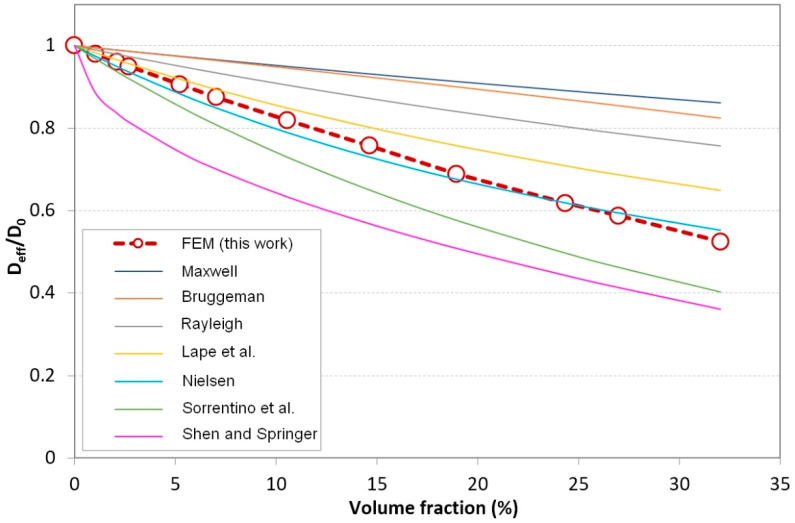
Comparison between simulation results and analytical models [8,43,44,45,46,48,49] from the literature (monodisperse fillers: *D* = 35 nm, *L* = 177 nm, *α* = 5).

Figure 4 shows the FEM-predicted evolution of the relative effective diffusivity with the filler volume fraction as well as the selected analytical model predictions. As expected, the barrier properties increase significantly with the volume fraction—the effective diffusivity is reduced by 30% for a volume fraction of about 18%, while for a loading content of 33%, the effective diffusivity is divided by 2.

The analytical equations yielding the closest predictions to our FEM results were those of Nielsen, Lape et al., and Sorrentino et al. These three models consider ribbon-like fillers and account for the aspect ratio dependency in addition to the volume fraction. Unsurprisingly, the equations proposed by Maxwell and Bruggeman for regular sphere arrangements led to noticeably slower decreases of the effective diffusivity with the increasing volume fraction.

Among these equations, it appeared that in the present case (aspect ratio *L*/*D* of the order of 5), the best agreement was provided by Nielsen’s model; however, it should be noted that this equation was derived by considering 2D diffusion in a regular array of ribbons with infinite perpendicular extension, which means that the tortuosity was mainly controlled by the parameter *L*; thus, when applied to our configurations, the predictions were expected to be very sensitive to the cylinder aspect ratio value and significant deviations could be observed with different rod lengths. On the contrary, Lape’s model is based on a random array of ribbons with an aspect ratio definition independent of the filler length, which is more similar to the configurations studied here. Although the diffusivity predictions of Lape’s model seem less accurate, they were expected to be more reliable when varying the filler *L*/*D* ratio, since they only depend on the cylinder cross section and volume fraction. This was proven to be true for our FEM simulations, as shown in the next section. 

### 3.2. Effects of Filler Aspect Ratio and Polydispersity

**Figure 5:**
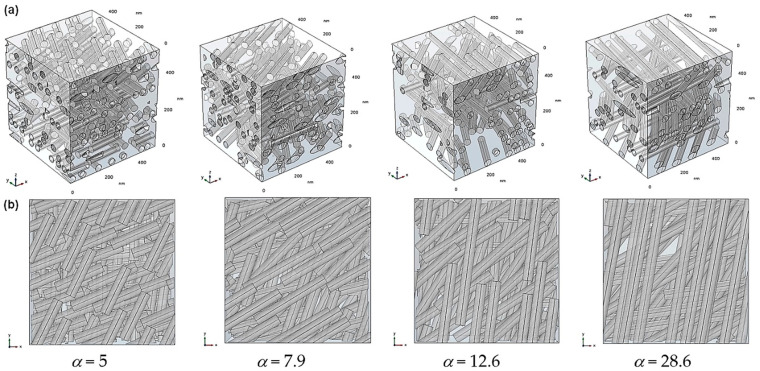
View of generated RVEs for several nanofiller aspect ratio values (monodisperse, volume fraction ≅ 22%): (**a**) isometric view; (**b**) projected view in the overall diffusion direction *z*.

In order to evaluate the influence of the nanorod aspect ratio on the composite diffusivity in the case of the 2-phase model, simulations were carried out using RVEs containing dispersions of monodisperse cylinders with a diameter *D* of 35 nm and increasing lengths *L* of 177, 278, 443, and 1000 nm. These values correspond to increasing aspect ratios *α* of 5, 7.9, 12.6, and 28.6. The value *L* = 1000 nm was chosen to be superior to the diagonal of the square *x*-*y* cross-section of the simulation domain (equal to 707 nm) in order to obtain a number of continuous rods across the domain. Note that these aspect ratio values are upper bounds—if a cylinder is cut by one of the RVE edges, then its apparent aspect ratio will be lower. Figure 5 shows examples of RVEs generated for a filler volume fraction equal to 22%. The projected views in the *z* direction (“top” views) show that fillers are well dispersed in the simulation domain and that in all cases the existence of straight paths available for the molecules to diffuse in is very limited.

**Figure 6:**
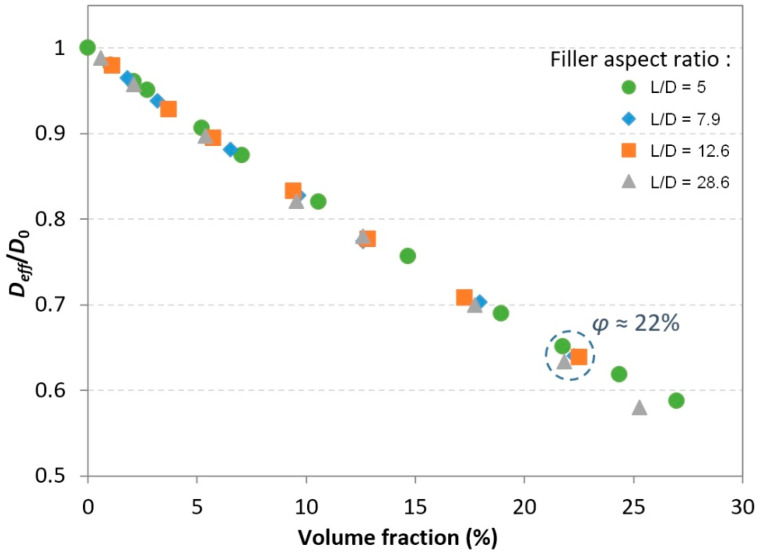
Effects of the filler aspect ratio on the relative effective diffusivity for monodisperse fillers.

The calculated relative effective diffusivity values are plotted in Figure 6. It can clearly be seen that for volume fraction values below 20%, the filler aspect ratio has a quite negligible effect on the barrier properties. Indeed, for a cylindrical filler shape, the length of the filler has very little effect on the tortuosity, since diffusing molecules tend to gather around the circumference of the rod rather than being forced to reach free spaces between the ends of two neighboring rods (which would result in a much longer diffusion path). 

Beyond this 20% value, only a slight deviation towards lower diffusivity values can be observed for the highest aspect ratio fillers (i.e., quasi-continuous rods). In this case, due to the lengthy fillers, the free spaces between the ends of neighboring fillers are sparser and located very far away from each other, which tends to marginally increase the tortuosity with respect to that of shorter cylinders. Nevertheless, this trend was not observable for the intermediate aspect ratio values. 

**Figure 7:**
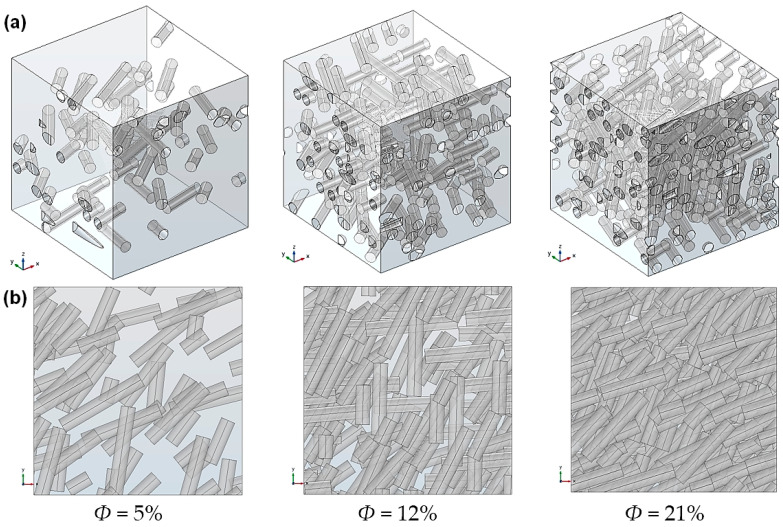
View of generated RVEs with polydisperse uniform dispersions for several volume fraction values (average aspect ratio $\bar{\alpha }=4.3$): (**a**) isometric view; (**b**) projected view in the overall diffusion direction *z*.

The influence of the filler length polydispersity was also investigated. For a given target volume fraction value, nanorod dispersions were randomly generated with length values following a uniform distribution in the 50–250 nm range. According to this distribution, each value in this range has an equivalent probability of appearance. It has been verified that for several generated distributions containing the same number of fillers, the generation algorithm does not favor a particular sub-interval in the desired length range, i.e., there is no trend of generating more short or long fillers in order to attain the target volume fraction. The average aspect ratio of these uniform distributions is then $\bar{\alpha }=4.3$. Figure 7 shows examples of RVEs generated for three different volume fraction values of 5%, 12%, and 21%. 

**Figure 8:**
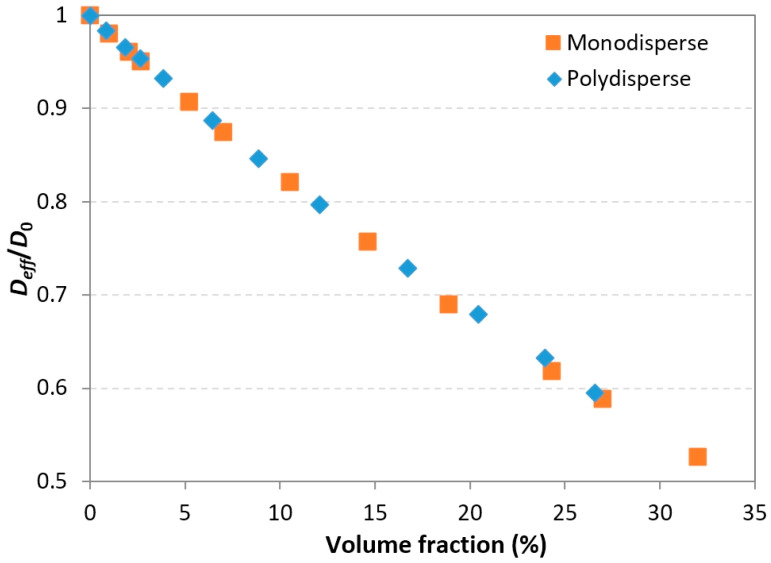
Effects of filler length polydispersity on the relative effective diffusivity (polydisperse: uniform length distribution in the 50–250 nm range, average aspect ratio $\bar{\alpha }=4.3$).

Simulations with the 2-phase model were performed for polydisperse systems with volume fractions of up to 26%. The calculated relative effective diffusivity values are represented and compared to those of monodisperse systems in the same range in Figure 8. No significant differences were found between the monodisperse and polydisperse populations. This was expected, since it had been demonstrated previously (Figure 6) that the filler length has no significant influence on effective diffusivity; hence, it can be concluded that for polydisperse nanorods, although some regions contain shorter fillers, there is no overall reduction of the system tortuosity or diffusion path length. These results are consistent with the studies by Lape and al. [49] and Chen and Papathanasiou [50]. In Lape’s study, the considered fillers had flake-like shapes and it was shown that the barrier properties were even increased regarding the polydispersity. This trend was not observed in the present study, since the influence of the cylinder length on the tortuosity was not as apparent as that of the disk diameter. Additionally, it should be noted that in the study by Chen and Papathanasiou, a slight deviation was observed only for the higher filler loading content.

### 3.3. Effects of Filler Agglomeration

Filler agglomeration may be observed in the morphologies of nanorod-filled composites. This phenomenon is generally unwanted and can originate from the nanocomposite preparation process itself, from the nanofiller storage conditions prior to incorporation within the matrix, or from particular surface properties preventing good dispersion of fillers in the matrix. On the other hand, filler agglomeration could also be a deliberate way to modify the apparent aspect ratio of the fillers perpendicularly to the diffusion direction in order to promote barrier properties. In the following paragraphs, simplified models of agglomerated nanorods dispersions are studied and their diffusion properties are assessed with respect to that of “ideal” dispersions of perfectly isolated fillers.

The simplified model is as follows. Agglomerated fillers are generated as clusters of three identical and parallel cylinders in line contact with each other. Mass diffusion is not possible between contacting cylinders belonging to the same cluster. The axes of the three cylinders belong to the same *x*-*y* plane, meaning that the clusters are not tilted and they remain perpendicular to the overall diffusion direction. The random positioning of the clusters follows the same procedure as the one used for the isolated fillers, as described in Section 2.2. The simulations were carried out with monodisperse cylinders, since we had previously shown that the influence of polydispersity is negligible. 

**Figure 9:**
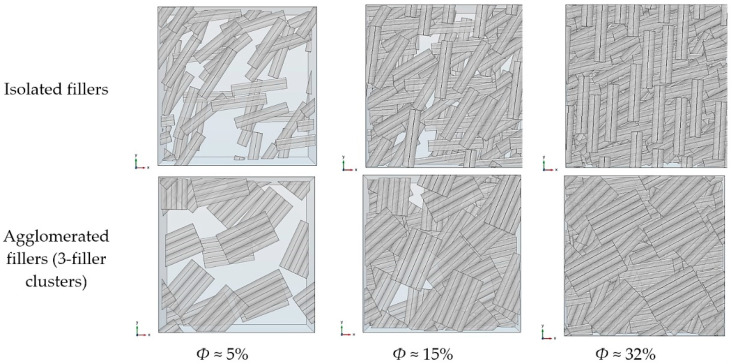
Projected view in the overall diffusion direction z of generated RVEs for isolated fillers and 3-filler clusters (agglomerated) for several volume fraction values.

Figure 9 shows projected views of generated RVEs for the reference case of isolated fillers and for agglomerated fillers with the same volume fraction values. It can be postulated that on one hand, agglomeration may increase the local tortuosity by increasing the filler aspect ratio, while on the other hand this may imply the existence of larger free, filler-less matrix regions, since the dispersed phase is distributed more heterogeneously over the whole RVE. These larger free regions could allow a more important diffusive flux, leading to an increase in overall diffusivity; hence, it can be expected that the effective diffusivity of the agglomerated systems will result from the opposite influence of both phenomena. 

**Figure 10:**
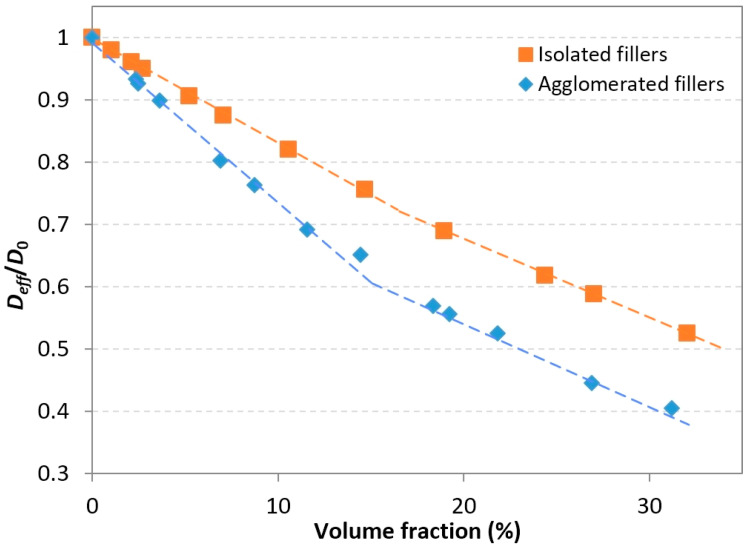
Effects of filler agglomeration (3-filler clusters) on the relative effective diffusivity (monodisperse fillers, *α* = 5). The dashed lines are guides.

In order to determine which one of these phenomena was dominant in this particular case, numerical simulations (2-phase model) were performed for agglomerated systems with volume fractions up to 32%. All generated cylinders had a diameter *D* = 35 nm and a length *L* = 177 nm (cylinder aspect ratio *α* = 5). The calculated relative effective diffusivity values are represented and compared to those of isolated fillers systems in the same range in Figure 10. 

The plot clearly shows that for any given volume fraction investigated, agglomerated fillers led to a lower effective diffusivity than isolated fillers. Below the 15% volume fraction, the deviation between the effective diffusivities of both systems increased with the filler volume fraction. For the 7% volume fraction, agglomeration reduced the diffusivity by 8.3%, while for the 20% volume fraction, the reduction was about 20%; however, for higher volume fraction values, the deviation did not increase as obviously. Indeed, for a 32% volume fraction, the improvement brought about by agglomeration (with respect to isolated fillers) was only 23.8%, which was not very far from the value obtained for the 20% volume fraction. This effect is visible in the overall shape of the plots; each curve can be described as following a bi-linear shape, with a slope change around the 15% volume fraction. In the 0–15% range, the slope of the plot corresponding to the agglomerated fillers is more pronounced than the one corresponding to the isolated fillers. Beyond 15%, the slopes of both plots are equivalent. 

These results show that the barrier effect induced by the agglomerated fillers due to increasing local tortuosity is the most prevalent effect here. Inevitably, agglomeration also results in increased distances between fillers, which tends to degrade barrier properties. Nonetheless, this effect seems to be of less importance for small agglomerates, such as those simulated here. Finally, it is reiterated that this model is a simplified representation of actual systems, in which agglomerates could have variable sizes (variations in length, width, and thickness); accordingly, they may be either flake-like (thin and wide aggregates, such as the 3-rods clusters used here) or block-like (width and thickness of the same order), which would result in different levels of influence on the tortuosity and effective diffusivity.

### 3.4. Effects of Filler–Matrix Interphase Properties

Two-phase models such as the one developed in the previous sections of this study have been widely used to predict the barrier properties of composites. Our model is to some extent an ideal representation. Several experimental observations showed that the matrix area located near the filler surface could be considered as a third phase with specific properties. This is the so-called filler–matrix interphase. The existence of this phase can be attributed to several causes. It may result from poor adhesion between the hydrophobic polymer matrix and hydrophilic particles [51,52], creating voids within the matrix in the area surrounding the filler. For fillers with surface modifications based either on ionic exchange or grafting of molecules of different chain lengths and mobilities, the existence and significant role played by this third phase on the barrier properties have been experimentally proven [38,53,54,55,56,57,58,59]. In some cases, the barrier properties are enhanced by the presence of the interphase due to stronger filler–matrix interfacial interactions [32,35,60,61,62]. In other cases, increased mobility or a lack of packing density in the interphase region lead to degradation of the barrier effect [51,63,64,65]. 

A number of models describing the interphase’s influence on transport properties are available in the literature. Such models are based on geometrical and analytical approaches [8,66,67,68] or numerical approaches, mainly at the mesoscale using FEM [42,69] or at smaller scales using molecular dynamics [70,71,72,73,74]. FEM-based models basically consider the interphase as a third medium (or phase) surrounding the fillers, with well-defined boundaries and homogeneous diffusion properties. In this framework, two key modeling parameters govern the influence of the interphase on the barrier properties—the interphase diffusivity and the interphase thickness. The main difficulty with these parameters is that they are not easily accessible by direct experimental characterization; however, their influence can be evaluated through parametric studies.

#### 3.4.1. Effects of the Interphase Diffusivity

In this section, a wide range of interphase diffusivity values is considered, from a virtually impermeable interphase to a highly diffusive interphase. The relative interphase diffusivity parameter *k* is defined as the ratio of the interphase diffusivity to the matrix diffusivity:

(8) $$k=\frac{D_{int}}{D_{0}}$$

**Figure 11:**
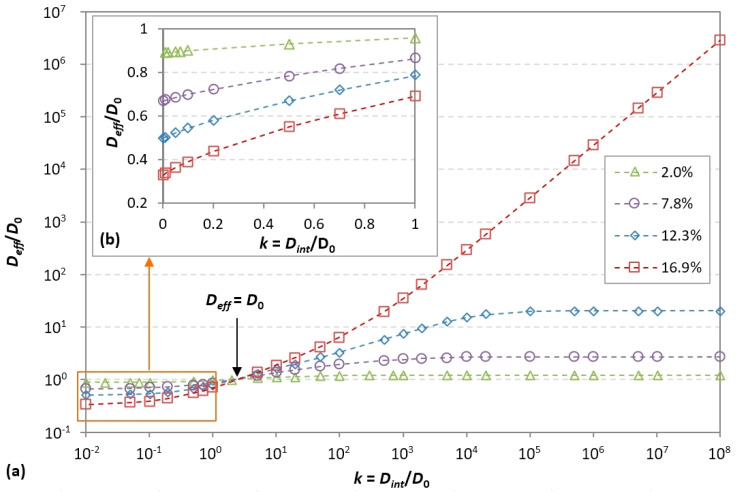
Effects of filler–matrix interphase diffusivity on the nanocomposite’s relative effective diffusivity for several filler volume fractions (2 %, 7.8 %, 12.3 % and 16.9 %), with an interphase thickness *e_int_* = 10 nm: (**a**) full *D_int_* range (log scale); (**b**) *D_int_* < *D*_0_ (linear scale).

Simulations were carried out with *k* values ranging between 10^−2^ and 10^8^ for 4 randomly generated monodisperse systems (fillers *D* = 35 nm, *L* = 177 nm) with volume fractions values of 2%, 7.8%, 12.3%, and 16.9%. The dimensions of the RVEs were *L_x_* = *L_y_* = 500 nm and *L_z_* = 620 nm, with 15 layers of fillers along the *z*-direction. The spacing between layers was 40 nm. The thickness of the interphase surrounding each filler was set to *e_int_* = 10 nm. This value corresponds to an interphase thickness-to-filler-thickness ratio of 0.285, which is within the usual range of values reported in the literature [75,76,77,78]. According to these conditions, if two fillers are close enough to each other (in a given *x*-*y* plane or in adjacent layers along the *z*-direction), their interphases can overlap and form a continuous phase around these fillers; hence, for sufficient volume fraction values, continuous diffusion paths may appear, the length of which may be much greater than the filler size. Depending on the interphase diffusivity, such a phenomenon may have a significant impact on the barrier properties. The calculated relative effective diffusivity values versus relative interphase diffusivity values are shown in Figure 11. 

Overall, it can clearly be seen that the influence of the interphases is all the more significant as the filler volume fraction increases. All curves intersect at a common point corresponding to *D_eff_*/*D*_0_ = 1, i.e., the nanocomposite’s effective diffusivity is equal to the neat matrix diffusivity. In the present case, this point occurs for an interphase diffusivity of about 3 times the matrix diffusivity. At this particular point, which could be named the “iso-diffusive” point, the tortuosity effect induced by the fillers is exactly counterbalanced by the higher diffusion rate in the interphase regions than in the matrix. It should be noted that the location of this point is dependent on the interphase thickness, as shown in [42]; hence, under these particular conditions (resulting from increased mobility or a lack of packing density in the interfacial regions), the barrier effect brought about by the fillers can simply be annihilated and there is no more effect of the loading content on the barrier properties.

For lower interphase diffusivity values (i.e., below the iso-diffusive point), all four curves exhibit similar behavior, whereby the effective diffusivity decreases as the interphase diffusivity decreases. This can be interpreted as an apparent increase of the filler volume fraction, since in this case the interphase behavior tends towards impermeability, as do the fillers themselves. Additionally, the asymptotic trend reveals that this barrier-enhancing effect reaches a limitation as the interphase diffusivity becomes low enough (below 5 × 10^−2^), which is to be expected. Figure 11b presents a close-up of the plots for interphase diffusivity values inferior to the neat matrix diffusivity. As an example, for a filler volume fraction of 16.9% and an interphase diffusivity value equal to 10% of the matrix diffusivity, the achieved composite’s relative effective diffusivity is reduced to 0.4 (compared to 0.65 if interphases are not taken into account, i.e., *D_int_*/*D*_0_ = 1). 

For higher interphase diffusivity values (i.e., beyond the iso-diffusive point), there is a significant degradation of the barrier properties. Two contrasting types of behavior are observed, whereby the curves corresponding to the three lower volume fractions 2.0%, 7.8%, and 12.3% all exhibit similar sigmoidal shapes in the log-log scale, while the 16.9% curve shows a continuous rise when the interphase diffusivity increases. 

In the first case, the effective diffusivity increases with the interphase diffusivity for *D_int_* values up to 10^5^, beyond which an asymptotic trend is reached. The maximum effective diffusivity is equal to 20 times the matrix diffusivity for the 12.3% volume fraction. This type of behavior can be associated with interphases becoming more diffusive and occasionally overlapping but remaining disconnected from each other overall, which prevents the formation of highly diffusive pathways at large scales; hence, the degradation of the barrier properties remains limited.

In the second case (illustrated by the 16.9% curve), the asymptotic behavior is not present and the effective relative diffusivity increases linearly with the interphase diffusivity, which means there is no limit to the degradation of barrier properties. This can be explained by the appearance of large-scale continuous diffusion paths within the interphase. As the interphase diffusivity becomes higher, the diffusion resistance of these paths becomes lower and the flux of permeating species is preferentially attracted to the interphase network instead of diffusing within the matrix. The observed linear behavior is consistent with the physics of diffusion and Fick’s law in a homogeneous medium; for a given concentration difference, the diffusive flux is proportional to the diffusivity of the medium. Indeed, if continuous paths are created over a length scale of the same order as the RVE thickness and if the interphase diffusivity is sufficiently high compared to the matrix diffusivity, diffusion will virtually occur only along these continuous paths, “ignoring” the matrix phase. This corresponds exactly to the case of diffusion in a single homogeneous medium. One should note that for random positioning of fillers, such a configuration is not always obtained for the same exact filler volume fraction. Regarding barrier properties, this is the worst case that can be encountered, as the resistance to gas diffusion can be drastically and uncontrollably lowered if the quality of the filler–matrix interfaces is not sufficient. 

#### 3.4.2. Effects of the Interphase Thickness

**Figure 12:**
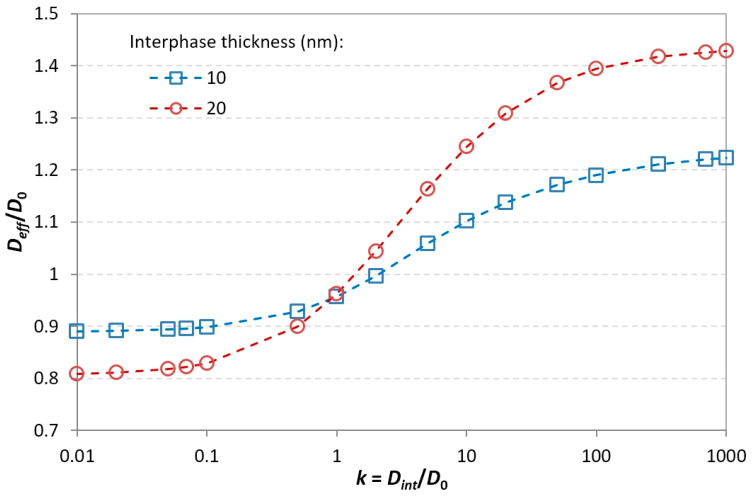
Effects of the filler–matrix interphase thickness and diffusivity on the nanocomposite’s relative effective diffusivity for 2% filler volume fraction.

The other modeling parameter for the interphase in the FEM framework is the interphase thickness *e_int_*. For cylindrical fillers (diameter *D*, length *L*), the matrix–interphase boundary is a cylinder with diameter *D_int_* = *D* + 2*e_int_* and length *L_int_* = *L* + 2*e_int_*. In order to study the influence of this parameter on the evolution of the effective diffusivity, simulations were carried out on monodisperse systems with a filler volume fraction of 2%, for interphase thickness values of 10 and 20 nm. The interphase’s relative diffusivity *k* values were limited to the range for which the most significant variation of effective diffusivity is observed, i.e., 0.01–1000. The obtained relative effective diffusivity values versus relative interphase diffusivity values are plotted in Figure 12. 

As expected, the effects of the interphases are more significant if their thickness is larger. Obviously, both curves intersect for *k* = 1, which is equivalent to the absence of interphase. With respect to the 10 nm interphase case, increasing the interphase thickness by a factor of 2 results at most in a 11% lower effective diffusivity for the less diffusive interphase and a 16% higher effective diffusivity for the more diffusive interphase. The interpretation of this behavior is similar to the interpretation of the influence of the interphase diffusivity. The theory of Fickian transport states that the diffusive flux is proportional to the cross-section normal to the direction of diffusion; thus, an increase in the interphase thickness increases the diffusion rate by the same factor under a given local concentration gradient. Moreover, thicker interphases tend to enhance the probability of interphases overlapping and the formation of continuous diffusion paths, which have been shown to be detrimental to the barrier properties. 

## 4. Conclusions

In this study, a 3D numerical model of gas diffusion in polymer nanocomposite systems filled with nanorods was implemented and solved with the finite element method under the Fickian diffusion assumption. The simulated systems were representative of poly(lactic acid)–cellulose nanocrystal composites, which are very attractive bio-based materials for packaging and coating applications thanks to their O_2_ and CO_2_ barrier properties. The model allowed the investigation of the effects of several key morphological parameters on the nanocomposite barrier properties, as quantified by the relative effective diffusivity. The two-phase model considers only the matrix and the fillers, whereby the filler amount is clearly the most influential parameter; however, there is virtually no effect of the filler aspect ratio or of the filler length polydispersity on the barrier properties, since the system tortuosity is not significantly affected by an increase in the nanorods lengths. The effect of filler agglomeration was assessed by considering systems filled with typical clusters of 3 nanorods. For a sufficient volume fraction, the effective diffusivity was found to be significantly lower for agglomerates than for isolated fillers. The three-phase model takes into account the existence of filler–matrix interfacial regions with specific properties. According to the simulation results, the effective diffusivity is strongly affected by the presence of interphases, especially as the filler amount is higher. Depending on the interphase quality (nearly impermeable or highly diffusive), this effect can be either beneficial or detrimental to the barrier properties. Moreover, for sufficient filler volume fractions and highly diffusive interphases, the interconnection of neighboring interphases may lead to the formation of continuous diffusion paths at a large scale through the nanocomposite, which is especially critical for the barrier properties. The thickness of the interphase also plays a role by governing the interphase diffusion section, i.e., thicker interphases tend to amplify either the barrier enhancement (for impermeable interphases) or the barrier degradation (for highly diffusive interphases). 

The parametric studies that were carried out were very useful in order to understand the influence of the nanocomposite key parameters on the transport properties. The model will be used to provide guidelines for the preparation of films with optimal properties. The next steps will include the comparison of simulation results to experimental measurements on PLA–CNC films with different types of CNC surface chemical modifications, as well as the extension and adaptation of this modeling approach to systems containing higher filler volume fractions. In such cases, the filler “network” could be considered as the continuous phase, whereas the matrix would play the role of the dispersed phase.

## Figures and Tables

**Table 1:** Analytical equations predicting the relative effective diffusivity values of composite systems.

Model	Filler Shape	Distribution	Equation, $\frac{D_{\mathrm{eff}}}{D_{0}}$	Ref.
Maxwell	Spheres	Regular	$\frac{1}{1+\phi /2}$	[43]
Bruggeman	Spheres	Regular	${\left(1-\phi \right)}^{1/2}$	[44]
Rayleigh	Fibers	Regular	$\frac{1}{1+\phi }$	[45]
Shen and Springer	Fibers	Regular	$1-2\sqrt{\frac{\phi }{\pi }}$	[46]
Nielsen	Ribbons	Regular	$\frac{1}{1+\frac{\alpha \phi }{2}}$	[8]
Sorrentino et al.	Ribbons	Random	$\frac{1}{1+\frac{4\phi }{\pi \left(1-\phi \right)}\left(1+\frac{1}{\beta }+\frac{\beta }{4}\right)}$	[48]
Lape et al.	Ribbons	Random	$\frac{1}{{\left(1+\frac{\beta \phi }{3}\right)}^{2}}$	[49]

## Data Availability

The data presented in this study are available on request from the corresponding author.
